# Iron Starvation Conditions Upregulate *Ehrlichia ruminantium* Type IV Secretion System, *tr1* Transcription Factor and *map1* Genes Family through the Master Regulatory Protein ErxR

**DOI:** 10.3389/fcimb.2017.00535

**Published:** 2018-01-19

**Authors:** Amal Moumène, Silvina Gonzalez-Rizzo, Thierry Lefrançois, Nathalie Vachiéry, Damien F. Meyer

**Affiliations:** ^1^Centre de Coopération Internationale en Recherche Agronomique Pour le Développement, UMR ASTRE, Petit-Bourg, France; ^2^ASTRE, Univ Montpellier, Centre de Coopération Internationale en Recherche Agronomique Pour le Développement, Institut National de la Recherche Agronomique, Montpellier, France; ^3^UFR Sciences Exactes et Naturelles, Université des Antilles, Pointe-à-Pitre, France; ^4^Institut de Biologie Paris Seine (EPS - IBPS), Sorbonne Universités, UPMC Univ Paris 06, Univ Antilles, Univ Nice Sophia Antipolis, Centre National de la Recherche Scientifique Evolution Paris Seine, Paris, France; ^5^Equipe Biologie de la Mangrove, UFR Sciences Exactes et Naturelles, Université des Antilles, Pointe-à-Pitre, France

**Keywords:** *Ehrlichia ruminantium*, master regulator, iron regulation, T4SS, *map* genes, *tr1* transcription factor, environmental cues

## Abstract

*Ehrlichia ruminantium* is an obligatory intracellular bacterium that causes heartwater, a fatal disease in ruminants. Due to its intracellular nature, *E. ruminantium* requires a set of specific virulence factors, such as the type IV secretion system (T4SS), and outer membrane proteins (Map proteins) in order to avoid and subvert the host's immune response. Several studies have been conducted to understand the regulation of the T4SS or outer membrane proteins, in *Ehrlichia*, but no integrated approach has been used to understand the regulation of *Ehrlichia* pathogenicity determinants in response to environmental cues. Iron is known to be a key nutrient for bacterial growth both in the environment and within hosts. In this study, we experimentally demonstrated the regulation of *virB, map1*, and *tr1* genes by the newly identified master regulator ErxR (for *Ehrlichia ruminantium* e*x*pression *r*egulator). We also analyzed the effect of iron depletion on the expression of *erxR* gene*, tr1* transcription factor, T4SS and *map1* genes clusters in *E. ruminantium*. We show that exposure of *E. ruminantium* to iron starvation induces *erxR* and subsequently *tr1, virB*, and *map1* genes. Our results reveal tight co-regulation of T4SS and *map1* genes via the ErxR regulatory protein at the transcriptional level, and, for the first time link *map* genes to the virulence function *sensu stricto*, thereby advancing our understanding of *Ehrlichia's* infection process. These results suggest that *Ehrlichia* is able to sense changes in iron concentrations in the environment and to regulate the expression of virulence factors accordingly.

## Introduction

*Ehrlichia ruminantium*, the causal agent of heartwater, a fatal disease of ruminants in sub-Saharan African and other tropical regions, belongs to the *Anaplasmataceae* family and is transmitted by ticks of the genus *Amblyomma* (Dumler et al., 2001; Allsopp, 2010). In the mammalian host, *E. ruminantium* mainly infects brain capillary endothelial cells and replicates inside membrane-bound vacuoles (Zweygarth and Josemans, 2001). *E. ruminantium* has a biphasic developmental cycle in which elementary bodies (EB), the infectious form of the bacterium, first adhere and enter host cells. After internalization, EB differentiate into reticulate bodies (RB), the vegetative and non-infectious form, which divide by binary fission. Within 4–5 days, RB reorganize into EB, which are released from the vacuole by the lysis of the host cell to initiate a new infectious cycle (Moumène and Meyer, 2015).

Intracellular pathogenic bacteria belonging to the *Anaplasmataceae* family such as *Ehrlichia*, use a dedicated system, the type IV secretion system (T4SS), to inject some bacterial proteins, named effectors, to evade the host's immune responses and to hijack host cell processes in order to survive and proliferate in a safe replicative niche (Moumène and Meyer, 2015). T4SS is well conserved in the *Anaplasmataceae* family and several T4SS effectors (T4Es) have been shown to be crucial for the pathogenicity of *Anaplasma phagocytophilum, Anaplasma marginale*, and *Ehrlichia chaffeensis* (as reviewed by Rikihisa, 2010). Little is known about T4SS in *E. ruminantium* and no T4Es have yet been characterized (Collins et al., 2005; Frutos et al., 2007).

In several bacteria, it has been shown that the expression of T4SS is tightly regulated by transcription factors (Li and Carlow, 2012; Martín-Martín et al., 2012). Cheng et al. (2008) showed that the five *virB/D4* genetic loci of *E. chaffeensis* T4SS are co-regulated by the transcription factor EcxR to allow specific expression, depending on the developmental stage of the bacteria (Cheng et al., 2008). Previous studies have also demonstrated developmental regulation of the expression of T4SS components during the intracellular life cycle of *A. phagocytophilum* in human peripheral blood neutrophils (Niu et al., 2006). A *ecxR* ortholog, *apxR*, has been found in several *A. phagocytophilum* strains (Wang et al., 2007a,b). ApxR regulates the expression of the transcription factor *tr1* (Wang et al., 2007b) and of the downstream *p44E* locus (Wang et al., 2007a). Interestingly, the *virB/D4* loci of *A. phagocytophilum, E. chaffensis*, and *E. canis* are flanked by genes encoding outer membrane proteins (OMP) belonging to the *p44/msp2* family, which are paralogs of *Ehrlichia ruminantium map1* genes (Dunning Hotopp et al., 2006; Rikihisa, 2011). The exact environmental cues, which stimulate the expression of *apxR* and *ecxR*, are not known.

Considering all these observations, we analyzed the genome of *E. ruminantium* and found an ortholog of *ecxR* and *apxR* in *E. ruminantium*, hereafter termed *erxR*, for the *Ehrlichia ruminantium* e*x*pression *r*egulator. Moreover, as we observed in *A. phagocytophilum, tr1* is present in *E. ruminantium* upstream of the cluster of *map1* OMPs (Wang et al., 2007b). In *E. ruminantium*, the 16 paralogs of the *map1* multigene family are expressed in bovine endothelial cells and some are preferentially transcribed in the tick or in the mammalian host (van Heerden et al., 2004). However, whether or not ErxR is a regulatory protein, which drives the expression of *tr1* gene, the *map1* genes family, and the *virB/D4* loci in *E. ruminantium* and the triggering stimuli are currently unknown.

Microorganisms have evolved sensory mechanisms to regulate their cellular activities in response to environmental changes. This is particularly true for bacterial pathogens whose expression of virulence factors is tightly regulated in response to host and non-host environments (Hyytiäinen et al., 2003). Thus, the regulation of T4SS in response to host cues enables the efficient use of bacterial resources and facilitates colonization, leading to full infection (Abromaitis et al., 2013). One such environmental signal is iron, which is an essential cofactor in various enzymatic reactions like respiration, DNA replication, oxygen transport, response to oxidative stress, but can be toxic at excessive intracellular concentrations (Andrews et al., 2003). Therefore, iron scavenging from the limited sources of free iron available in the host is a crucial determinant of bacterial pathogenicity (Ratledge and Dover, 2000). Pathogens have evolved several ways to scavenge iron from the host, including the expression of iron acquisition genes in response to low iron concentrations (Brickman et al., 2011; Portier et al., 2014). In Gram negative bacteria, TonB-dependent outer membrane receptors (TBDR) are required to transfer iron chelates and heme into the periplasm under poor iron conditions, with subsequent transport to the cytoplasm (Shultis et al., 2006). Whether Map1 proteins play a similar role in *E. ruminantium's* iron uptake or sensing is currently not known.

In this study, we consequently investigated the effect of the newly identified ErxR protein and iron conditions on the regulation of the *virB/D4, tr1* and *map* genes expression in *E. ruminantium*. For the first time in *Ehrlichia*, we identified (i) a master regulatory protein responsible for the coordinated regulation of the expression of *virB/D4, tr1*, and *map* genes, and (ii) one triggering environmental signal i.e., iron depletion, which activates this regulation cascade. Our work also demonstrates that *map* genes share at least one regulatory pathway with genes encoding the T4SS and may therefore be important pathogenicity determinants for iron acquisition or host cell infection.

## Materials and methods

### Culture conditions

*E. ruminantium* Gardel strain (passages 30 to 52) was routinely propagated in bovine aortic endothelial (BAE) cells as previously described (Marcelino et al., 2005). To evaluate the growth characteristics of *E. ruminantium* under iron depletion conditions, the strain was grown in TC25 cm^2^ flasks in BHK-21 cell medium supplemented with 2 mM glutamine, 10% heat inactivated fotal bovine serum (FBS), penicillin (100 IU/ml), streptomycin (100 mg/ml). For iron response experiments, the medium was supplemented with iron (100 μM FeSO_4_) or an iron chelator (100 μM 2,2′-bipyridyl; BPD) as described in Breuer et al. (1995) and Romeo et al. (2001). The cells were kept in a humidified atmosphere supplemented with 5% CO_2_ at 37°C. FeSO_4_ or 2,2′-bipyridyl was added when 80% cell lysis was observed, at 120 h post-inoculation (hpi). The *E. ruminantium* infected cell monolayer (1 TC25 cm^2^ flask) was harvested by trypsinisation 24 h after chemical inoculation. From the 6 ml of infected cell supernatant, a 600 μl sample was collected by centrifugation at 20,000 × g for 10 min. The pellet was stored at −80°C until DNA extraction. The remaining 5,400 μl of infected cells were centrifuged at 20,000 × g for 10 min. The pellet was resuspended in TRIzol reagent (Invitrogen) and stored at −80°C until RNA extraction (Pruneau et al., 2012).

### Quantitative detection of *E. ruminantium*

Genomic DNA was extracted from the 600 μl samples described above using the QIAamp DNA Mini Kit (Qiagen, France). The number of bacteria per sample was quantified by q-PCR, targeting the single copy of *map1* gene encoding a major antigenic protein. The primer sequences are shown in Supplementary Table 1 (Pruneau et al., 2012). A standard curve was established using gDNA of Gardel serially diluted from 7 × 10^6^ to 7 × 10^1^ copies μL^−^1, to determine the number of bacteria per microliter (Pruneau et al., 2012). Four microliters were added to Taqman master mix (Applied Biosystems, France), following the manufacturer's instructions. PCR conditions were as follows: 2 min at 50°C, 10 min at 95°C, and 40 cycles with 15 s at 95°C and 1 min at 60°C.

### Relative gene expression: RNA preparation and qRT-PCR

Total RNA was extracted using TRIzol reagent. RNA pellets were dissolved in 100 μl of DEPC water and treated with turboDNAse (Ambion, France). The purity and concentration of the isolated RNA were assessed using a NanoDrop 2000c (Thermo Scientific, France). RNA samples were diluted in RNase-free water to obtain a final concentration of 0.5 μg/μL. RNA samples were reverse-transcribed with the SuperScript VILO cDNA Synthesis Kit (Invitrogen, France), according to the manufacturer's instructions. Quantitative PCR was performed in a 7500 Real-Time PCR System (Applied Biosystems, France) using a Power SYBR Green PCR Master Mix (Applied Biosystems, France) and the primers listed in Supplementary Table 2. Reactions were performed in 25 μl volume with 5 ng template cDNA and 5 μM of each primer. The amplification conditions were as follows: 2 min at 50°C, 10 min at 95°C and 40 PCR cycles (30 s at 95°C and 1 min at 60°C). An additional dissociation step of 15 s at 95°C, 20 s at 60°C and 15 s at 95°C was added to assess non-specific amplification. A negative control without cDNA template was included for each primer combination. Amplifications were performed in technical replicates consisting of independent cDNA syntheses derived from the same RNA sample and in three independent biological replicates. The relative expression of *erxR* was calculated by dividing the number of transcripts by the total number of bacteria at each time point. Fold change was then calculated by comparing the relative expression at each time point and the relative expression at 96 hpi, the stationary phase. Ratios were calculated from the number of transcripts and normalized to *recA* as described in Gonzalez-Rizzo et al. (2006).

### Identification of orthologs of EcxR in the *E. ruminantium* genome

To identify orthologs of *E. chaffeensis ecxR* in the genome of *E. ruminantium*, we used the same strategy as that described in Li and Carlow (2012). The protein sequence of ECH_0795 (YP_507593) was used as a query to search the genome of *E. ruminantium*. Multiple sequence alignment was performed using ClustalW (http://www.ebi.ac.uk/Tools/msa/clustalw2/) (Larkin et al., 2007). Sequence identity values between the two sequences were generated using BlastP. A Helix-Turn-Helix motif was determined using Pfam (https://www.ebi.ac.uk/Tools/hmmer/search/hmmscan) to find domains and motifs present in the protein sequence.

### Cloning and expression of *erxR*

Full-length *erxR* was PCR amplified using the primers listed in Supplementary Table 2, and ligated into the *Nde*I and *Xho*I sites of the pET29a(+) vector (Novagen). The resulting plasmid, defined herein as pErxR, was cloned into *E. coli* DH5α (Invitrogen) for amplification, purified using the QIAGEN Plasmid Maxi Kit (Qiagen, France), and cloned into *E. coli* BL21 (DE3) (Invitrogen) for protein expression. Protein expression was induced with 4 mM isopro-pyl-β-D-thiogalactopyranoside (IPTG) in 250 ml terrific broth. The protein then was purified using the Ni-NTA Fast Start Kit (Qiagen, France). ErxR expression was determined by Western blot analysis using anti-His tag antibody (Qiagen, France).

### Construction of pUA66-derived promoter plasmids

The pUA66 plasmid was used to analyse promoter activity based on the expression of a green fluorescent protein (GFP). The promoter regions were PCR amplified from the genomic DNA of *Ehrlichia ruminantium* Gardel strain, using the primers listed in Supplementary Table 2. Forward and reverse primers contained *Hind*III and *BamH*I restriction sites for cloning into the pET29a(+) plasmid. After cloning in pET29a(+), the promoters were digested with *Xho*I and *BamH*I for directed cloning into the pUA66 plasmid (Castaño-Cerezo et al., 2011). BL21 (DE3) cells were co-transformed with pErxR and each of the GFP reporter constructs, individually. The pET29a(+) vector alone was used as a negative control. Cotransformants were grown in LB medium supplemented with 50 μg/ml kanamycin at 37°C for 2 h, followed by induction with 1 mM IPTG for 4 h. Induced bacteria were visualized as described below.

### Microscopy

A 6-μl drop of *E. coli* BL21 (DE3) co-transformed with the pUA66 promoter containing one of the different promoters and the pET29a-erxR plasmid (Supplementary Table 3), resuspended in LB growth medium, was spotted onto a Superfrost Plus slide (Fisher Scientific Ltd, UK) and visualized using a Nikon Eclipse 80i epifluorescence microscope (Nikon, France). Fluorescent images were acquired with a Nikon DXM1200F digital camera (Nikon, France), using Nikon ACT-1 software (Nikon, France). Fluorescence intensity was calculated by measuring the area, integrated intensity and mean gray value of the fluorescent bacteria and the background with ImageJ (National Institute of Health, USA). Corrected total cell fluorescence (CTCF) was calculated using the following formula: integrated density—(area of the cell × mean background readings). The average and statistical differences between the bacteria containing the plasmids with the different promoters and controls were calculated using the CTCF values of all the bacteria in four different fields of view. Images were processed to size, and brightness and contrast were adjusted after the measurements, using Adobe Photoshop cs5 (Adobe Systems Inc., California, USA).

### Statistical analyses

Statistical analyses used Student's *t*-test and a *P* < 0.05 was considered significant.

## Results

### Identification of one *ecxR* ortholog in the *E. ruminantium* genome

**Figure 1:**
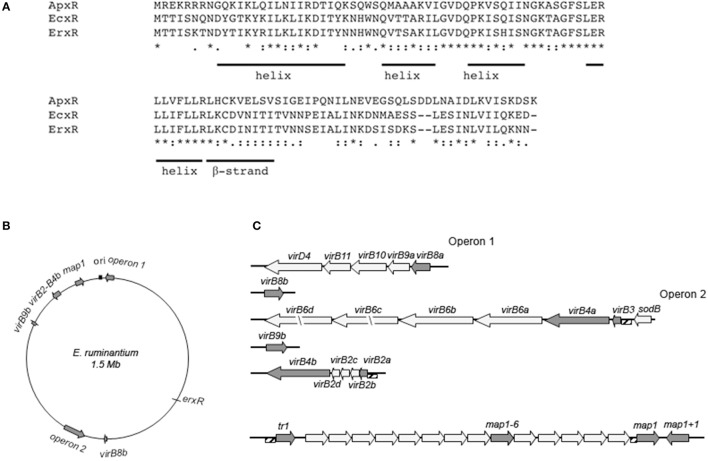
Potential ortholog of the *Ehrlichia* type IV secretion system regulator EcxR in *E. ruminantium* and organization of *virBD* loci and *map1* cluster. **(A)** Amino acid alignment of EcxR and its orthologs from *E. ruminantium* (ERGA_CDS_03000) and *A. phagocytophilum* (ApxR). Identical and conserved residues are indicated by stars and dots, respectively. The positions of amino acids predicted to form a helix structure and beta-strand are shown. **(B)** The genomic map of *E. ruminantium* is represented by a circle. The origin of replication (ori) is indicated by a black box. Gray arrows show the *virBD* gene loci and cluster *map1*. T4SS operon 1 consists in *virB8a, virB9a, virB10, virB11*, and *virD4* and operon 2 consists in *sodB, virB3, virB4a, virB6a, virB6b, virB6c*, and *virB6d*. Four copies of *virB2* were identified upstream of *virB4b*. *map1* genes belong to a multigenic family organized in one cluster of 16 paralogs located downstream of the transcriptional factor *tr1*. The length of the arrows is proportional to the length of the gene. **(C)** Cluster structure of *virBD* gene loci and *map1* genes. *virBD* and *map1* genes whose expression under iron starvation condition was tested and are represented by gray arrows. The names and length of the genes are indicated above and below the arrows. The upstream regions amplified by PCR for regulation assays (hatched boxes) are indicated.

We used the *ecxR* sequence (YP_507593.1) from *E. chaffeensis* to search NCBI databases using the BLAST tool. With this approach, we identified ERGA_CDS_03000 (YP_196226.1) in the genome of *E. ruminantium* as the closest ortholog to *ecxR*. The results of the BLAST search revealed a putative conserved domain belonging to the HXT_XRE superfamily of DNA binding proteins (cl17200). We identified a helix-turn-helix structure by comparing it with the structure found in *Wolbachia* (Li and Carlow, 2012) and further confirmed by Pfam (https://www.ebi.ac.uk/Tools/hmmer/search/hmmscan). This protein comprising 124 amino acids has a predicted molecular mass of 14.25 kDa. Alignment of the deduced amino acid sequences of the various orthologs is shown in Figure 1A. By homology with ApxR and EcxR, we named this protein *E. ruminantium* expression regulator, ErxR. Comparison of the sequence identities of these proteins revealed a high degree of conservation (82% identity) between EcxR and ErxR. ApxR and ErxR showed 40% identity at the amino acid level. Structural analyses indicated that all orthologs shared a conserved helix-turn-helix domain that may function as a sequence specific DNA binding domain, such as in transcription regulators (Aravind et al., 2005).

### Architecture of T4SS and *map1* genes clusters of *E. ruminantium*

We compared the genetic arrangement of *E. ruminantium* to that of *E. chaffeensis*. The five *virBD* loci are represented in Figures 1B,C. In *E. ruminantium*, the genome sequence revealed the presence of two major operons. *virD4, virB11, virB10, virB9a*, and *virB8a* were located in operon 1. Operon 2 was seen to be located in the negative strand and contained four copies of *virB6*, along with one copy of *virB4a* and *virB3*, all located downstream of *sodB*. Four duplicated versions of *virB2* were also located upstream of *virB4b*, while copies of *virB8* and *virB9* (namely *virBb8* and *virB9b*) were scattered along the genome. We also present the arrangement of the *map1* gene family previously reported by Postigo et al. (2007) (Figure 1C).

### Analysis of *erxR* expression during the life cycle of *E. ruminantium*

**Figure 2:**
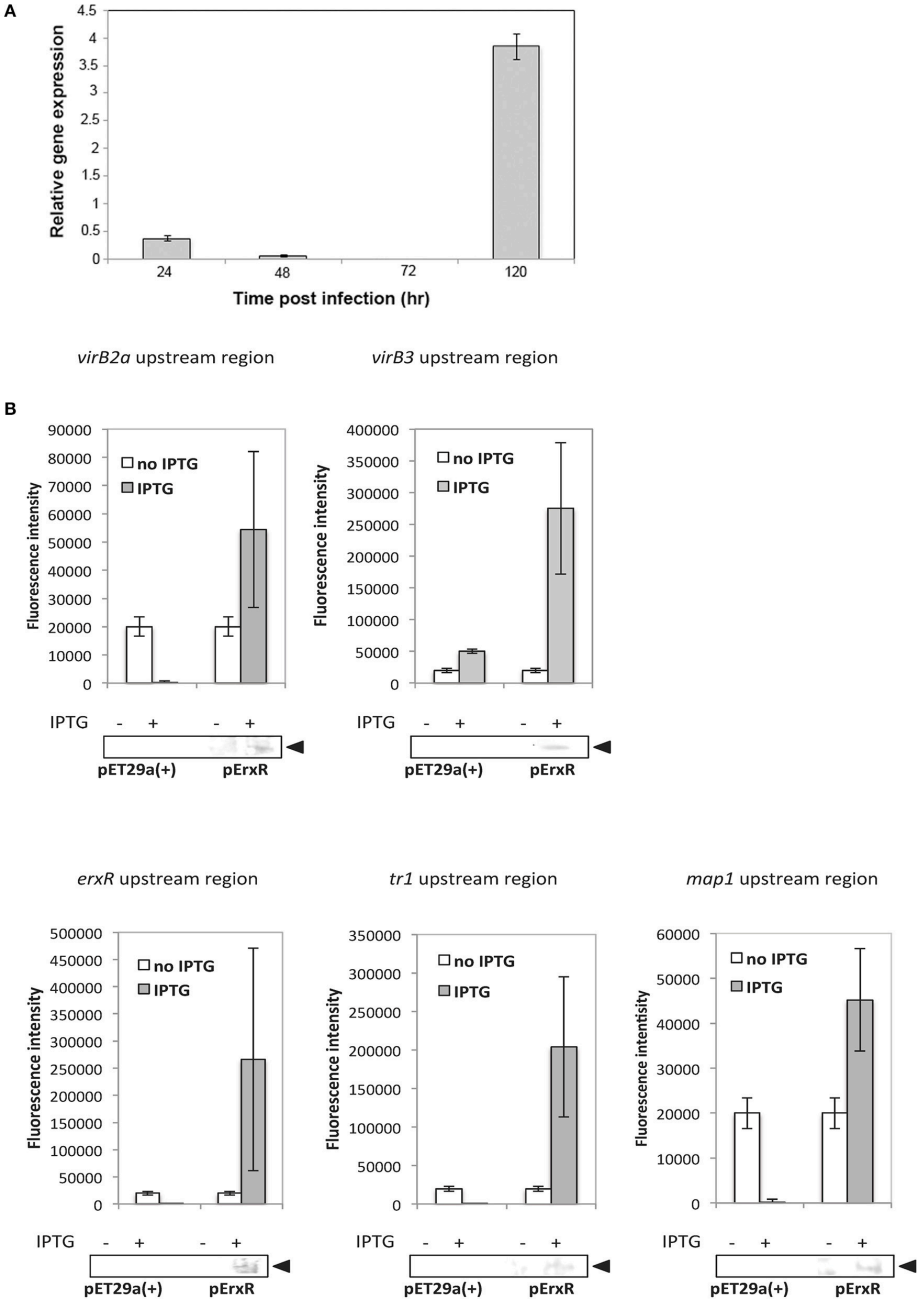
**(A)** *erxR* expression peaks at 120 hpi in *E. ruminantium*. Quantitative RT-PCR was used to determine the temporal expression of *erxR*. Relative expression at different developmental stages was normalized by dividing the transcripts number by the number of bacteria. Fold differences were evaluated by comparing each time point to 96 hpi (the stationary phase). Data were obtained from triplicate samples and are expressed as means + standard deviation. **(B)** ErxR activates the transcription of *virBD, tr1* and *map* genes. Fluorescence intensity was used to measure the transcriptional activities of *gfp* reporter constructs. The values are means + standard deviations for three specimens, and measurements were taken from four different view fields. An asterisk indicates that values differ significantly (*P* < 0.001) from the controls. Western blot analyses were performed of samples from the fluorescence assays using an anti-His antibody to verify the expression of rErxR. Arrowheads indicate the position of rErxR.

To determine the relative expression of *erxR* throughout the developmental cycle of *E. ruminantium, erxR* mRNA expression was analyzed by qRT-PCR. Compared to 96 hpi, the expression of *erxR* decreased at 24, 48, and 72 hpi, and peaked at 120 hpi, having increased 4 fold (Figure 2A), which corresponds to the time of lysis (early time point in the following round of infection). Data from previous independent microarray experiments (Pruneau et al., 2012) also demonstrated that the maximal expression of *erxR* in the post-exponential growth phase is significant and reproducible (data not shown). These results suggest that the up-regulation expression of *erxR* correlates with early stages in the development cycle *in vitro* before the bacteria enter the host cell.

### rErxR activates *gfp* reporter fusions

**Figure 3:**
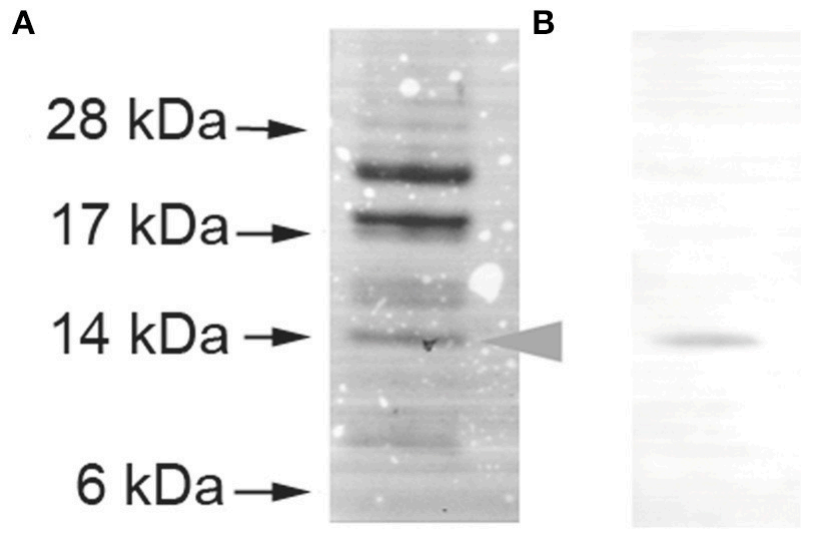
Production of rErxR in *E. coli*. *E. ruminantium erxR* was cloned into the pET29a(+) vector, expressed, and purified using nickel chelate chromatography. The purified protein was subjected to SDS- PAGE analysis, followed by Coomassie blue staining **(A)** and Western blot analysis using an anti-His tag antibody **(B)**. We used pre-stained protein size standards. Each lane contained 8 μg of recombinant protein.

The promoter regions (hatched boxes in Figure 1) for *virBD* genes, *map1, tr1*, and *erxR* were cloned into a *gfp* reporter plasmid and transformed into *E. coli* BL21 (DE3) carrying pErxR or an empty pET29a(+) vector to investigate if ErxR regulatory protein drove their expression. The *virB3*-*gfp* reporter constructs presented a significant increase in fluorescence intensity after IPTG induction (270,000 units of fluorescence intensity) compared to samples lacking IPTG (10,000 units of fluorescence) or compared to the control (50,000 units of fluorescence) (Figure 2B). Similarly, the reporter construct showed an induction of *virB2a* by the recombinant ErxR protein. Activation was also observed for *tr1* (200,000 units), *erxR* promoter (250,000), and *map1* promoter (~45,000) (Figure 2B). Western blot experiments confirmed that the expression of ErxR (14 kDa band) was only detected following induction with IPTG (Figure 3).

### The expression of T4SS, *erxR, tr1*, and *map* genes is induced by iron depletion in *E. ruminantium*

**Figure 4:**
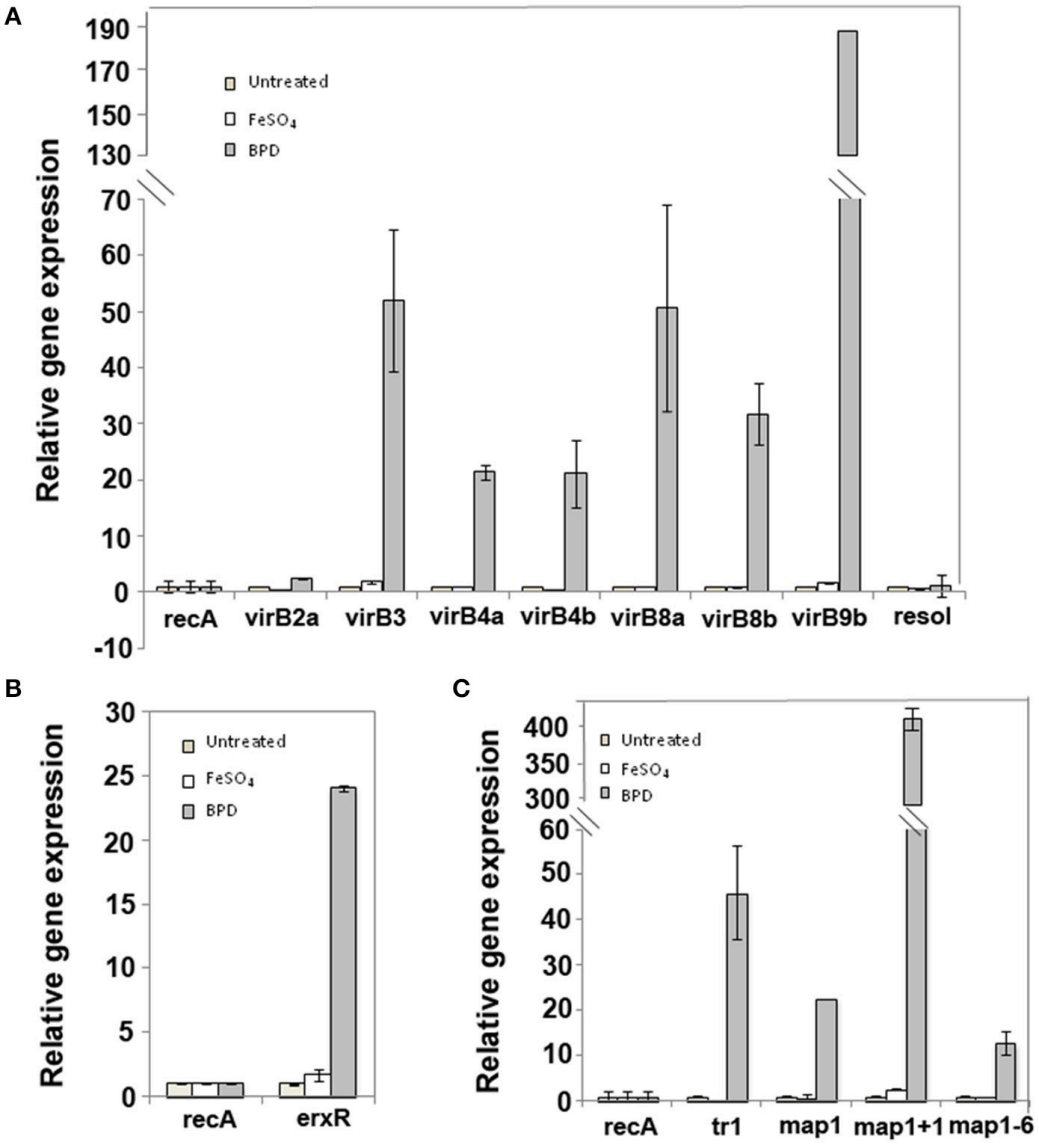
*virBD, tr1* and *map* genes as well as *erxR* are up-regulated under iron-depletion. The expression of the *virB* **(A)**, *erxR* **(B)**, *tr1* and *map1* **(C)** genes were measured during the lysis phase of infection under iron repletion or iron depletion using quantitative real-time PCR. The data represent the mean + SD of 2 or 3 biological replicates, each of which comprised 3 technical replicates. Ratios were calculated from the transcript numbers and normalized to *recA*. The *resol* gene was used as negative control.

As iron uptake mechanisms are closely associated with bacterial pathogenesis and may be connected to the expression of certain virulence determinants in *E. ruminantium*, we investigated the expression of T4SS, *tr1* and *map* genes clusters in response to iron starvation. We incubated the bacteria in media containing iron or an iron chelator (BPD) for 24 h after lysis. The five T4SS transcription units of *E. ruminantium* were significantly up-regulated under iron depletion (25 to 190 fold increase as shown for *virB3, virB4a, virB4b, virB8a, virB8b*, and *virB9b*) (Figure 4A). *virB2a* was the only gene that did not present a significant change in expression (Figure 4A). Moreover, the *erxR* gene was up-regulated 25 fold during iron starvation (Figure 4B). We tested the expression of the *map1* gene cluster under iron starvation in the two genes at the border of the cluster (*tr1* and *map1*+*1*) as well as in the archetypal *map1* and *map1-6, which* are located in a central position in the cluster. Culture under iron limitation strongly increased the expression of all genes (Figure 4C), however *map1*+*1* showed the highest change in expression with 400-fold up-regulation (Figure 4C), suggesting that this gene may play a key role during iron starvation.

## Discussion

The preferential expression of virulence factors with diverse functions in response to host and environmental cues has already been characterized in several bacteria (Oogai et al., 2011; Weber et al., 2014). Thus, several *Ehrlichia* and other members of the *Anaplasmataceae* family have been shown to differentially express certain genes in response to the host cells (Singu et al., 2006; Nelson et al., 2008).

These genes include genes encoding components of the T4SS, which have been shown to play an essential role in pathogenicity (Rikihisa, 2010; Moumène and Meyer, 2015). Many intracellular bacterial pathogens use T4SS to deliver effector molecules, which subvert the eukaryotic host cell defenses and other cellular processes to their own advantage (Trokter et al., 2014). The genetic arrangement of the 18 genes encoding the T4SS apparatus in *E. ruminantium* T4SS genes resembles that of *E. chaffeensis* (Figure 1).

Likewise, OMPs are often regulated by environmental signals and play an important role in bacterial pathogenesis by enhancing the ability of the bacteria to adapt to different environments (Blanvillain et al., 2007). Interestingly, T4SS affects outer membrane properties, which might be important for the adaptation of *Brucella* to growth both *in vitro* and *in vivo* (Wang et al., 2010). Herein, we have described the effects of one environmental cue, iron concentration, on the expression of the transcriptional regulator *erxR*, the components of the T4SS apparatus and members of the Map1 family during *E. ruminantium* infection cycle of BAE cells.

Regulatory proteins are known to play an important role in the survival and persistence of intracellular pathogens in their host (Cheng et al., 2008). EcxR (ECH_0795) is the only transcriptional regulator to be associated with the expression of T4SS components in *E. chaffeensis* (Cheng et al., 2008). In *E. ruminantium*, we identified *erxR*, an orthologous gene of *ecxR* (Figure 1A), by sequence homology. ErxR (*Ehrlichia ruminantium expression regulator*) binds and regulates its own promoter and the promoters of some *virBD* genes. These results show that ErxR is the regulatory protein of the T4SS of *E. ruminantium*. Using qRT-PCR at different time points, we also show that *erxR* is strongly expressed at an elementary body stage (120 hpi), like that demonstrated in *E. chaffeensis* (Cheng et al., 2008).

*ErxR* orthologs in other *Anaplasmataceae* also appear to be associated with the expression of important antigenic OMPs. Thus, ApxR regulates the transcription of *p44* transcription by binding to the *tr1* promoter during *A. phagocytophilum* infection of mammalian host cells (Wang et al., 2007a,b). *p44E* encodes the immunodominant pleomorphic 44-kDa major surface protein, which shows homology with the Map1 family in *E. ruminantium* as well as the P30 family in *E. canis* and the P28 family in *E. chaffeensis* (Dunning Hotopp et al., 2006). The major antigenic protein Map1 is part of a multigene family containing 16 paralogs tandemly organized in a head to tail arrangement that are located downstream of a hypothetical transcriptional regulator gene (*tr1*) (Postigo et al., 2007; Figure 1C), a similar arrangement to that reported for *p44, p30*, and *p28* in the other *Anaplasmataceae* (Dunning Hotopp et al., 2006). *tr1* is one of the three promoters identified in the *p44* expression locus and shown to be the strongest promoter driving the expression of a polycistronic mRNA containing OMP1, *p44ESup*, and *p44* (Barbet et al., 2005). *tr1* harbors a winged helix-turn-helix and a DNA binding motif and its part of the xenobiotic response element family of transcriptional regulators. However, the function of *tr1* remains unclear (Nelson et al., 2008), and whether or not *tr1* drives the expression of a polycistronic tandem mRNA containing several *map* homologs is still not known. According to our results, ErxR binds to *tr1* and to *map1* promoters (Figure 3). It is thus possible that it regulates the expression of the *map1* members in a similar way to *tr1* in *A. phagocytophilum* for *p44* expression.

Taken together, our results show that ErxR binds to *tr1, map1*, and *virBD* promoters, suggesting for the first time coordinated regulation of T4SS and OMP in *Anaplasmataceae*. Interestingly, in the endosymbiotic bacterium *Wolbachia* (wBm) wBmxR1 and wBmxR2, which are orthologs of ErxR, have been shown to co-regulate genes of the T4SS and riboflavin biosynthesis pathway (Li and Carlow, 2012). Riboflavin is an important co-factor for the survival of the endosymbiont's host, the filarial parasite *Brugia malayi* (Li and Carlow, 2012).

Next, we investigated whether the regulation of genes encoding the T4SS and members of the Map1 family by ErxR could be triggered by environmental and nutritional cues in the host cell. Our transcriptional analysis showed that *erxR, virBD, tr1*, and *map1* genes were upregulated in response to iron starvation (Figure 4). Interestingly, the fact that *virB2a* is not expressed under iron depletion could be due to a functional redundancy depending on the environment (*e.g*. mammalian host, vector cell, etc.) as previously shown for PopF1 and PopF2 proteins of the T3SS of *Ralstonia solanacearum* (Meyer et al., 2006). Similarly, *A. phagocytophilum* appears to express specific *virB2* paralogs in a host cell dependent manner as well as differential expression of *virB2* gene in tick cells or in human cells (Nelson et al., 2008). Many bacterial pathogens sense iron depletion as a signal indicating that they are within a vertebrate host (Skaar, 2010).

**Figure 5:**
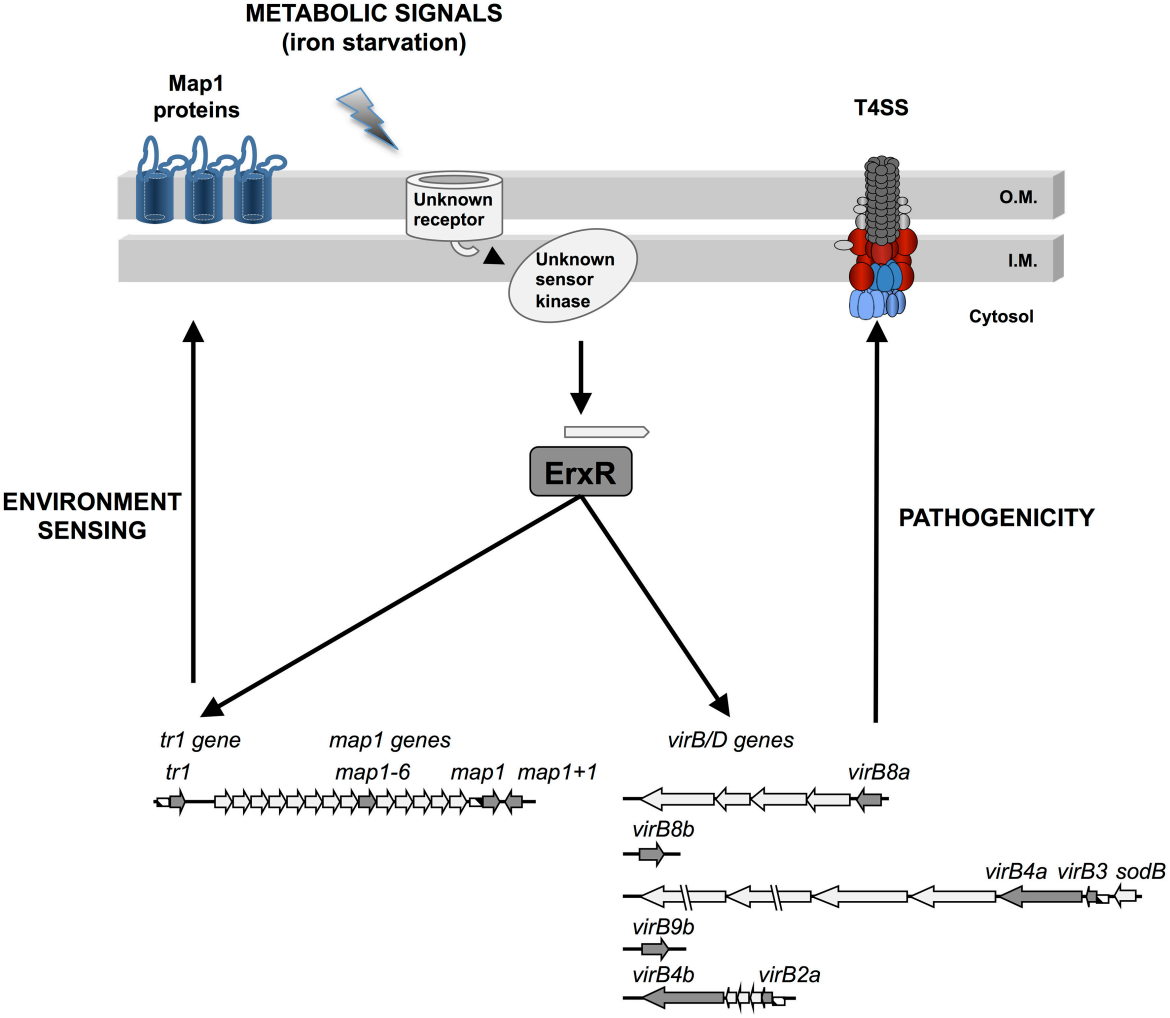
Putative regulation model for ErxR, *virBD, tr1* and *map* genes in *E. ruminantium* under iron starvation conditions.

Although the sensing mechanisms of changes in iron concentration are not known in *E. ruminantium*, three pathways are possible. One possibility is that the regulator ErxR is activated by an unidentified sensor kinase which responds to this environmental signal and up-regulates expression of the T4SS genes and certain *map1* genes in response to iron starvation (Figure 5). Sensor kinases are part of two component systems (TCS), which regulate the differential expression of genes in bacteria in response to environmental cues. Three TCS composed of three response regulators and three sensor kinases have been identified in *A. phagocytophilum* and *E. chaffeensis* (Cheng et al., 2006). All three sets of TCS have orthologs in *E. ruminantium* and one of these TCS could thus be involved in the regulation of pathogenic factors in response to changes in iron abundance.

Another possibility is the presence of an unknown Fur repressor in the *Anaplasmataceae* family. This sensing involves transcriptional control mediated by the transcriptional repressor, Fur (Escolar et al., 1998). But many organisms, including *E. coli, Campylobacter jejuni*, and *Vibrio cholera*, have been shown to use Fur to negatively regulate gene expression with increasing concentrations of iron (O'Sullivan et al., 1994; Palyada et al., 2004; Mey et al., 2005). For example, Fur activates *sodB*, an iron superoxide dismutase, under iron repletion. SODs are metalloproteins, which play an important role in protection against oxidative stress by catalyzing dismutation of the superoxide radical (${\text{O}}_{2}^{-}$). Interestingly, in *E. ruminantium, sodB* is located upstream of operon 2 of the T4SS and is co-transcribed along these genes in *Ehrlichia* and *Anaplasma*, suggesting an effect of iron in the expression of *virBD* genes. Moreover, it has been demonstrated that the regulator ApxR binds to the promoter regions upstream of *sodB* (Wang et al., 2007b). We searched for putative Fur boxes in the genome of *E. ruminantium* and found one located upstream of *erxR* (Supplementary Figure 1). The 19 bp sequence consisted in two repeated hexamers (nATWAT) flanking a 7 nt sequence, which is commonly found upstream of iron regulated enzymes such as succinate dehydrogenase iron-sulfur subunits, major ferric iron binding protein precursors, and adenosine tRNA methylthiotransferase (Escolar et al., 1998; Grifantini et al., 2003). Thus, it is possible that *E. ruminantium* is capable of sensing low iron concentrations in the environment and of regulating the expression of *ErxR* through this putative Fur box.

Finally, it is possible that some Map1 proteins play a role like that of TBDRs in the perception of environmental cues and in iron uptake (Blanvillain et al., 2007). As mentioned above, Map1 proteins are orthologs of members of the *p28* family in *E. chaffeensis*, which have been shown to function as porins and possibly act in nutrient uptake during intracellular infection (Kumagai et al., 2008). It has been shown that porins, such as OmpA and OmpC, bind to transferrin and act in iron uptake in the enteropathogenic strains of *E. coli, Salmonella typhimurium*, and several *Shigella* species (Sandrini et al., 2013). Similarly, *Mycobacterium smegmatis* is able to acquire ferric ions through members of the Msp family of porins (Jones and Niederweis, 2010). The up-regulation of *map1, map1*+*1*, and *map1-6* showed under iron starvation suggests that these three porins play a role in iron acquisition and raises the possibility that Map1 proteins may also act as sensors, although more evidence is needed before concluding. These results suggest that the Map1 proteins may fulfil several functions during *E. ruminantium* infection. Characterizing these functions could advance our understanding of the adaptation of *E. ruminantium* to its host, as done by Blanvillain et al. (2007).

In conclusion, we have demonstrated that exposure of *E. ruminantium* to iron limitation induces ErxR-dependent expression of the T4SS apparatus and *map1* genes. These findings reveal an important degree of coordination between T4SS and *map1* genes at the transcriptional level and raise the possibility of the involvement of Map proteins in environmental sensing and in the infection process. The data presented herein enables us to propose a model for the regulation of *E. ruminantium* T4SS and *map1* genes in which ErxR acts as a global regulator integrating iron as an important triggering environmental signal (Figure 5). Understanding *Ehrlichia* gene regulation in response to environmental signals provides valuable cues for the development of alternative treatments.

## Author contributions

DM, Conceived and designed the experiments. AM, SG-R, and DM, Performed the experiments. AM, SG-R, TL, NV, and DM, Analyzed the data. AM and DM, Wrote the paper.

### Conflict of interest statement

The authors declare that the research was conducted in the absence of any commercial or financial relationships that could be construed as a potential conflict of interest.

## Supplementary material

Supplementary Figure 1 (A) Nucleotide sequence of the predicted *erxR* promoter. The initiation codon of *erxR* is shown in capital letters at the end of the sequence. The sequence in red represents the nATWAT motif. The−35 and−10 boxes of the *erxR* promoter are capitalized. **(B)** The potential *erxR* Fur box is compared with the consensus *E. coli* Fur box. Conserved residues are shown in red.

Supplementary Table 1 Oligonucleotide primers used for qPCR and qRT-PCR.

Supplementary Table 2 Oligonucleotide primers used to construct plasmids.

Supplementary Table 3 Strains and plasmids used in this work.
