# Supplementary figures and images for: Iron Starvation Conditions Upregulate Ehrlichia ruminantium Type IV Secretion System, tr1 Transcription Factor and map1 Genes Family through the Master Regulatory Protein ErxR
Source: Front Cell Infect Microbiol. 2018 Jan 19;7:535. doi: 10.3389/fcimb.2017.00535 (PMC5780451; doi:10.3389/fcimb.2017.00535)

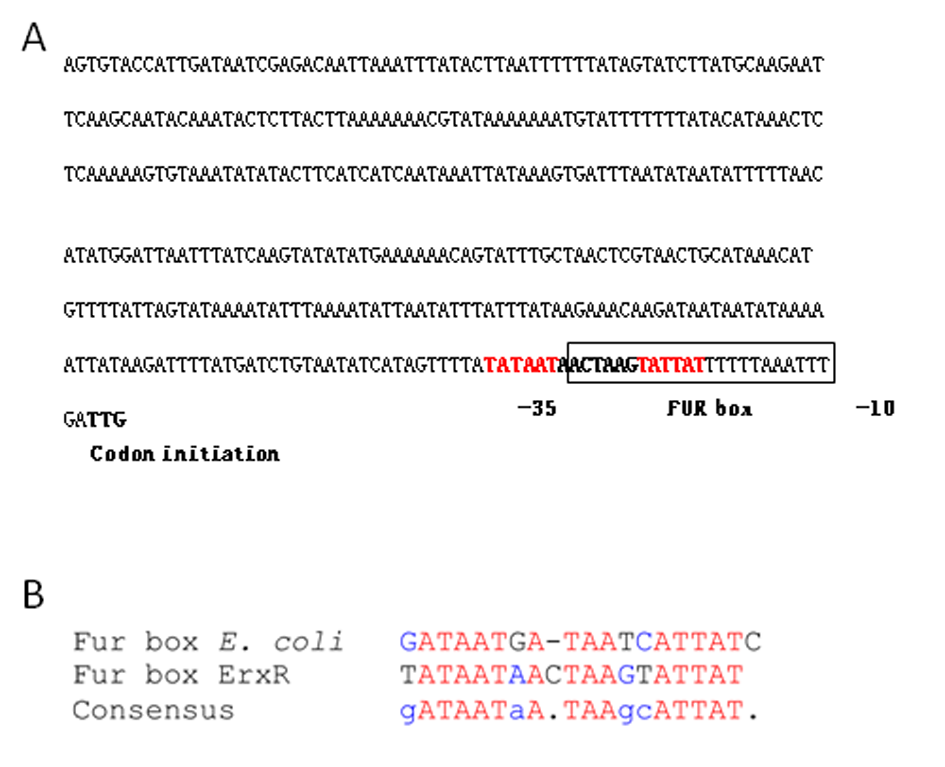

Supplement: Supplementary Figure 1 (A) — Nucleotide sequence of the predicted erxR promoter. The initiation codon of erxR is shown in capital letters at the end of the sequence. The sequence in red represents the nATWAT motif. The−35 and−10 boxes of the erxR promoter are capitalized. (B) The potential erxR Fur box is compared with the consensus E. coli Fur box. Conserved residues are shown in red. [file Image1.TIF]
