# Supplementary material for: Iron Starvation Conditions Upregulate Ehrlichia ruminantium Type IV Secretion System, tr1 Transcription Factor and map1 Genes Family through the Master Regulatory Protein ErxR
Source: Front Cell Infect Microbiol. 2018 Jan 19;7:535. doi: 10.3389/fcimb.2017.00535 (PMC5780451; doi:10.3389/fcimb.2017.00535)
Supplement: Supplementary Table 1 — Oligonucleotide primers used for qPCR and qRT-PCR. [file Table1.DOCX]

**Supplementary Table 1.** Oligonucleotide primers used for qPCR and qRT-PCR
