# Supplementary material for: Iron Starvation Conditions Upregulate Ehrlichia ruminantium Type IV Secretion System, tr1 Transcription Factor and map1 Genes Family through the Master Regulatory Protein ErxR
Source: Front Cell Infect Microbiol. 2018 Jan 19;7:535. doi: 10.3389/fcimb.2017.00535 (PMC5780451; doi:10.3389/fcimb.2017.00535)
Supplement: Supplementary Table 3 — Strains and plasmids used in this work. [file Table3.DOCX]

**Supplementary Table 3.** Strains and plasmids used in this work

| Strain | Properties | Source | |
| --- | --- | --- | --- |
| *Escherichia coli* DH5α | F- φ80*lac*ZΔM15 Δ(*lac*ZYA-*arg*F)U169 *rec*A1 *end*A1 *hsd*R17(rk^-^, mk^+^) *pho*A *sup*E44 *thi*-1 *gyr*A96 *rel*A1 λ- | Invitrogen | |
| *Escherichia coli* BL21 | - 1. F-*ompT hsdSB* (rB-mB-) *gal dcm rne131* (DE3) | Invitrogen | |
|  |  |  | |
| Plasmids |  |  | |
| pET29a(+) | Expression vector C-terminal His6x tag (Km^r^) | Novagen | |
| pUA66 | Promoter-probe plasmid using EGFP as a reporter of expression (Km^r^) | Castaño-Cerezo  *et al*., (2011) | |
| Km, kanamycin resistance | |  |  |
